# Gene Therapy for Inherited Metabolic Diseases

**DOI:** 10.34763/jmotherandchild.20202402si.2004.000009

**Published:** 2020-11-10

**Authors:** Berna Seker Yilmaz, Sonam Gurung, Dany Perocheau, John Counsell, Julien Baruteau

**Affiliations:** 1 Genetics and Genomic Medicine, Great Ormond Street Institute of Child Health, University College London, London, UK; 2 Department of Paediatric Metabolic Medicine, Faculty of Medicine, Mersin University, Mersin, Turkey; 3 Developmental Neurosciences Research and Teaching Department, Great Ormond Street Institute of Child Health, University College London, London, UK; 4 Metabolic Medicine Department, Great Ormond Street Hospital for Children, NHS Foundation Trust, London, UK; 5 National Institute of Health Research, Great Ormond Street Hospital Biomedical Research Centre, London, UK

**Keywords:** adeno-associated virus, gene therapy, inherited metabolic disease, lentivirus, messenger RNA, zinc finger nuclease

## Abstract

Over the last two decades, gene therapy has been successfully translated to many rare diseases. The number of clinical trials is rapidly expanding and some gene therapy products have now received market authorisation in the western world. Inherited metabolic diseases (IMD) are orphan diseases frequently associated with a severe debilitating phenotype with limited therapeutic perspective. Gene therapy is progressively becoming a disease-changing therapeutic option for these patients. In this review, we aim to summarise the development of this emerging field detailing the main gene therapy strategies, routes of administration, viral and non-viral vectors and gene editing tools. We discuss the respective advantages and pitfalls of these gene therapy strategies and review their application in IMD, providing examples of clinical trials with lentiviral or adeno-associated viral gene therapy vectors in rare diseases. The rapid development of the field and implementation of gene therapy as a realistic therapeutic option for various IMD in a short term also require a good knowledge and understanding of these technologies from physicians to counsel the patients at best.

## Introduction

Inherited metabolic diseases (IMD) are single gene disorders caused by enzymatic defects in metabolic pathways in which cumulative incidence is estimated as high as 1/800 (1, 2). Standard of care may include diet, enzyme and coenzyme replacement, removal of harmful substances, cell and organ transplantation and supportive therapies (3). In the past 20 years, gene therapy has emerged as a disease-changing treatment for these disorders (4).

Gene therapy is simple in principle, restoring normal cellular function by providing a functional copy of the defective gene by the addition of a new copy of the gene or tools to ‘edit’ the defective gene and correct the genetic mutation with specific vectors (5). Gene therapy had driven fantastic hope in the mid-1990s when addressing severe combined immunodeficiency (SCID) due to deficiency of the enzyme adenosine deaminase (ADA-SCID) (6). However, contemporary dramatic adverse events in historical clinical trials have tempered this enthusiasm when an ornithine transcarbamylase-deficient young adult died after a severe immune reaction against the gene therapy vector in a clinical trial (7) and leukaemias developed secondary to insertional mutagenesis in patients with X-linked SCID (X-SCID) (8). Subsequently, the research focused on safer delivery vectors and successful results have currently been reported for various inherited rare diseases such as Leber’s congenital amaurosis (9), X-linked adrenoleukodystrophy (10), metachromatic leukodystrophy (11), haemophilia B (12) and many other IMDs, leading to first market authorisations in Europe and in the USA in 2012 and 2017, respectively (13).

The strategies and delivery technologies developed to improve the efficacy and the safety of gene therapy are a field of intense research and interest. This review will outline the progress made from early stages to ongoing clinical trials by highlighting general principles and emphasising different delivery methods.

### General considerations

Gene therapy is the transfer or editing of a genetic material to cure a disease. Depending on the delivery strategy chosen, gene therapy can be performed *in vivo* or *ex vivo* with integrating (i.e. permanent modification of the host genome; e.g. lentiviral) or non-integrating (e.g. adeno-associated viral [AAV]) vectors (14).

### *In vivo* o*r ex vivo* gene therapy

*In vivo* gene therapy refers to the injection of a vector encoding the gene of interest or molecular tools for gene editing, directly into a tissue or into the systemic circulation to generate therapeutic outcomes in specific or multiple organs (15). *In vivo* gene therapy is based on the concept of providing an extra functional copy of the defective gene to slow or reverse the disease state (16). It often targets post-mitotic cells that are no more experiencing division (16). The main complications include non-specific targeting (also called off-target biodistribution) and immune responses to the vector (17).

*Ex vivo* gene therapy involves manipulation of a target cell population outside of the body, often as part of autologous stem cell therapies, in which a patient’s own cells are genetically modified with gene editing or gene supplementation and then engrafted back into the patient (18). *Ex vivo* therapy enables researchers to screen, isolate and expand the edited cells before re-administration. With specific protocols, researchers can eliminate the off-target cells and produce enough cells for transplantation (19). This strategy is limited to dividing cells. Some cell types are challenging to culture *in vitro* or have poor engraftment rate. For haematopoietic stem cells, this approach requires chemotherapy-based conditioning and its own risks (19). Integration means that the transgenic DNA becomes a permanent feature of the cell genome, being passed down to all future cell progeny. This is particularly attractive for treating dividing cells, such as stem cells and mitotically active paediatric tissues such as the paediatric liver.

### Genome editing

The gene therapy field was recently revolutionised by the introduction of genome editing tools, which includes nucleases engineered to modify the genome at precise loci. This includes zinc finger nucleases (ZFNs) (19), transcription activator-like effector nucleases (TALENs) (20), homing endonucleases (meganucleases) (16) and Clustered Regularly Interspaced Short Palindromic Repeats (CRISPR)/(CRISPR-associated system) Cas9 (16). These nucleases generate double-strand breaks in DNA, which is subsequently resolved by cellular DNA repair pathways. Two of these repair pathways are often exploited to mediate gene correction, addition and deletion or disruption: Non-Homologous End Joining (NHEJ) involves direct ligation of two DNA termini with intervening small sequence insertions and deletions (InDels), and Homology Directed Repair (HDR) achieves precise modification of the DNA through the introduction of a DSB along with an exogenous repair template. CRISPR/Cas9 is emerging as a popular tool for both *in vivo* and *ex vivo* gene therapy due to the diversity of Cas9 orthologues that have been developed and the readily customisable aspects of the platform, compared with other genome editing methods. Various proof of concepts have been developed for IMD in preclinical models (21, 22, 23). The main pitfalls are primarily the risk of introducing ‘off-target’ mutations in genome regions bearing similar sequence identity to the target site, which could produce an array of unwanted side effects, and recently it has been shown that up to 79% of tested human samples have pre-existing antibodies and 46% have T-cell responses against popular Cas9 orthologues, raising the risk of immune rejection (24). This has important implications if the intention is to deliver Cas9 long-term *in vivo* gene editing, although it has less relevance to *ex vivo* stem-cell therapies that are restricted to transient duration before transplantation.

### Immunogenicity

A massive obstacle to the translation of gene therapy is the high prevalence of immune response against the vectors, either pre-existing (25) when the patient has been previously exposed to the wild-type viral serotype used as gene therapy vector, or after exposure to the vector in the case of a re-injection is required. Viral vectors can evoke an innate immune response via several pathways, such as the sensing of pathogen-associated molecular patterns on vector particles or in the vector genome (26). While adenovirus (Ad) vectors provoke a robust innate immune response via complement activation, and both Toll-like receptor (TLR) TLR-dependent and TLR-independent mechanisms, lentiviral vectors cause an increase in the expression of several cytokines such as interferon (IFN)-a and IFN-β. Immune response against AAV vectors is known to be TLR3-independent, while humoral responses against the AAV capsid are enhanced by the presence of the complement (27). The recognition of these immune responses is not always identified during preclinical safety studies (4). Several approaches have been proposed to overcome this immune response: reducing the vector dose, capsid modifications, tissue-targeted gene transfer and immunomodulation (27).

In early onset IMDs, the deficient enzyme is often associated with a truncated native protein. The expression of the whole functional enzymatic protein can trigger an immune reaction against the transgenic protein itself. This has not been observed so far but remains a theoretical risk.

### Gene therapy in paediatrics

In most IMDs, an early treatment is essential to prevent debilitating complications, long-term sequelae and potentially death. An early administration can target more progenitor cells, which could theoretically enable a better effect for a same dose of gene therapy vector and can take advantage of an immature immune system. However, in rapidly growing organs such as the liver, non-integrative strategies of gene delivery means a progressive loss of the delivered transgene that will require re-injections, often precluded by a strong immune response triggered by the first injection (4).

Earlier, time for development of a promising gene therapy product—from the first patient injected in a clinical trial to market authorisation—has been slow from 8 to 16 years, although it is expected that the next products to be authorised might be developed at a quicker pace at present that the process has been successfully optimised with the first pioneering products (28).

### Gene therapy vectors

Various strategies to efficiently deliver nucleic acids to target organs have been developed over the last 30 years and are summarised with their respective advantages and pitfalls (Table 1).

**Table 1:** Main features of gene therapy strategies.

	Non viral	Lentivirus	Adenovirus	Adeno-associated virus
**Derived from pathogenic virus**	No	Yes	Yes	Yes
**Size of transgene**	No limit	14kb	7.5kb	4.7kb
**Insertion to host genome (Integration)**	No	Yes	No	Rarely
**Long-lasting gene expression**	No	Yes	Yes	Yes
**Safety issues**	No	Insertional mutagenesis	Immune response	Limited immune response

Kb : kilobases

### Lentiviral vectors

**Figure 1:**
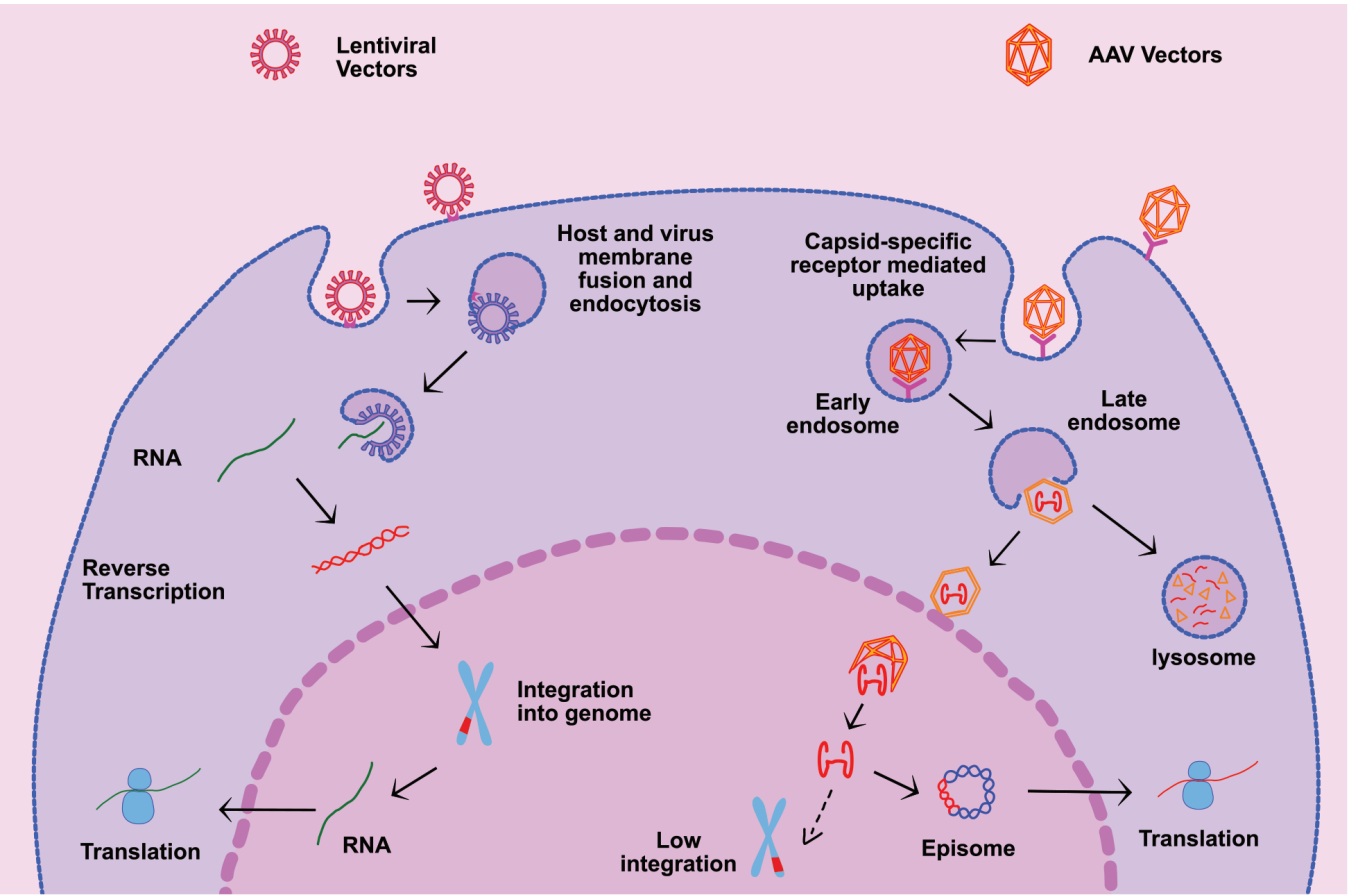
Transduction pathways of lentiviral and adeno-associated viral (AAV) vectors : cellular uptake and in-cell processing.

Lentiviral vectors, such as those based on human immunodeficiency virus type 1 (HIV-1), are a class of retrovirus that are used widely in gene therapy. Lentiviral vectors reverse-transcribe their single-stranded RNA genome on cell entry to form a double-stranded DNA product that translocates to the host nucleus and integrates into the host genome (Figure 1). Controversies over use of retroviral vectors have existed since induction of leukaemia in patients treated for X-SCID via *ex vivo* correction of autologous haematopoietic stem cells (HSC) (29). This X-SCID trial used a gamma-retroviral vector, which was prone to integrating in the vicinity of transcription start sites and enhancer elements (30), ultimately causing leukaemia in X-SCID patients. Lentiviral vectors are theoretically safer than gamma-retroviruses, as their integration patterns is less likely to interfere with endogenous transcriptional regulation (31). A key safety advance was achieved with development of the ‘self-inactivating’ (SIN) extremities of the lentiviral genome called long terminal repeat (LTR), designed to eliminate *cis*-acting enhancers in the retroviral LTR, the cause of oncogenesis in X-SCID patients (32). However, even with use of SIN-lentiviral technology, genotoxic adverse events have since been detected in subsequent lentiviral gene therapy trials (33). Lentiviral vectors have been engineered to mitigate this problem by reducing the content of native HIV-1 DNA in the delivered provirus, thereby eliminating problematic splice sites in the vector sequence (6).

In recent years, *ex-vivo* gene therapy trials involving SIN-lentiviral correction of HSC have shown strong clinical data in treating a range of severe monogenic childhood diseases, including X-SCID (34). Now, lentiviral vectors are increasingly moving into broader applications in gene therapy, including *ex vivo* manipulation of T cells, brain and mesenchymal stem cells and *in vivo* treatment of retina, brain, lung and liver. Recently, it has been shown that integration also provides a potential efficacy advantage over unintegrated vectors such as AAV (35). Additionally, for IMDs caused by large genes that are incompatible with AAV packaging capacity, such as carbamoyl-phosphate synthase deficiency, lentiviral vectors offer added value in their ability to handle larger payloads (36).

### Adenoviral vectors

Adenoviruses are non-enveloped double-stranded DNA viruses with a large 36 kb genome. They can target both dividing and non-dividing cells. Adenoviral serotypes are triggering a strong innate immune response (4). Adenoviral vectors are non-integrating vectors and their payload remains as circular DNA in the cellular nucleus. These vectors enable long-term expression and can accommodate large transgenes. The strong immunogenicity generated by capsid proteins was associated with a dramatic event in a clinical trial for OTC deficiency when a young patient died following a fulminant immune response and subsequent multiorgan failure (7). Other vectors with improved safety profile have been generated known as helper-dependent adenoviral vectors (HD-Ad) with deletion of most of the coding sequences from the adenoviral genome. The improved safety profile has shown benefit in preclinical studies and in clinical trials, but the acute innate immune response persists. This limits applications in IMD but is an advantage for its use in the field of cancer or vaccination (4).

### AAV vectors

AAV is a non-enveloped, single-stranded DNA parvovirus with a 4.7 kb genome, which is dependent on co-infection with a helper virus (like adenovirus) to replicate and generate a viral infection (37). It is composed of the *rep* and *cap* genes flanked by inverted terminal repeats (ITRs) (4). The *rep* gene encodes four proteins that are required for viral genome replication and packaging, while *cap* expression gives rise to the viral capsid proteins which protects the viral genome and acts in cell binding and internalisation (37). AAVs are uptaken through serotype-specific receptor/co-receptor at the surface of the target cell. An AAV receptor common to various serotypes facilitate intracellular trafficking (38). The transgene persists as episome in the nucleus as AAVs are mostly non-integrative but integration can occur at very low rate (Figure 1) (4).

Depending on serotypes, 20–80% of the general population has pre-existing antibodies against the capsid proteins, which is one of the major hurdles for clinical translation. These antibodies, even at low titres, are often neutralising the transduction of the target cell, especially if the vector is delivered in the bloodstream. As there is currently no validated immunosuppressive protocol, which has shown reasonable efficacy in mitigating this risk, clinical trials are recruiting seronegative patients for the AAV capsid used (25, 39).

Due to the small size of the genome and simple viral life cycle, AAV is emerging as one of the most successful gene therapy vectors, particularly with their safety and effectiveness in some monogenic disease trials (40). Several successful trials have been conducted for a variety of inherited diseases since the mid-2000s leading to the first gene therapy product receiving market authorisation in the West for lipoprotein lipase deficiency in 2012 (13).

### Liposomes

Liposomes are spherical vesicles enclosing an aqueous space within a synthetic lipid bilayer membrane used as non-viral gene therapy vectors (41, 42). Phospholipids and sphingolipids are commonly used lipids that allow their natural self-assembly (43). Their size varies from 20 nm in diameter to a few microns. They are particularly attractive gene therapy vector carriers as they can transport large pieces of DNA and protect genetic cargo from degradation. Cationic liposomes are more popular as they offer almost 100% loading efficiency. The negative charges in DNA interact with cationic liposomes to form complexes that allow increased DNA uptake (44, 45, 46). They are biocompatible and have low immunogenic risk and no replication risk (47). Their physiochemical and biophysical properties can be modified for drug loading and targeting to specific cells and tissues, such as they have also been used successfully to deliver genes to cells both *in vitro* and *in vivo* (48). *In vivo* work has mostly used antibody-based targeting strategies with reporter gene cargoes for application in cancer (49, 50, 51).

Their main challenges are a limited delivery with non-specific binding, reduced stability *in vivo*, limited efficacy caused by lysosomal degradation and limited ability to access nuclear compartments for DNA cargo (52, 53). Serum compounds can also interfere with their structure and promote aggregation, which can cause immunogenic responses and toxicity (54, 55).

### Exosomes

Exosomes are small vesicles (30–150 nm in size) naturally secreted by most cell types at the end of the endocytic pathway post-fusion of late endosomes/multivesicular bodies (MVBs) with the plasma membrane (56, 57) and represent another form of non-viral gene therapy vectors. Exosomes play a critical role in cell–cell communication by delivering functional proteins and genetic contents such as mRNA and miRNA transcripts to the recipient cells (58, 59). Hence, they influence both physiological and pathological processes in cells (60, 61).

Due to their natural ability to transfer genetic information, exosomes present themselves as an attractive target to be exploited for therapeutic application (62, 63, 64). They are biocompatible and are composed of non-viral components that reduce their immunogenic risk and ensure their longevity in circulation. Their bilayered lipid structure can protect the genetic cargo from degradation, and their small size and flexibility can facilitate them to cross major biological membranes including the blood–brain barrier (65, 66). They can be modified to enhance their targeting abilities to specific cells and tissues (67). The main challenges include robust and reproducible methods for exosomes manufacturing, characterisation and efficient cargo loading (68), immunogenicity (69) and a limited understanding of exosome biology (57, 70).

However, encouragingly, preclinical studies have shown the safety of multiple injections (71). While the use of exosomes in IMD remains at its early days, recent studies used engineered exosomes loaded with b-glucocerebrosidase (GBA) *in vitro* showing proof of concept with an increase in GBA activity (72, 73).

### Messenger RNA

The use of messenger RNA (mRNA) delivery for gene transfer is an appealing strategy as mRNA can be translated rapidly into protein once reaching the cytoplasm. mRNA provides temporary, half-life-dependent protein expression that allows adjustable protein production.

mRNA is often delivered via lipid nanoparticles (74, 75), a strategy explored in few IMDs (76, 77). LNPs are liposome-like structures encapsulating genetic materials like RNA and DNA but are made of single lipid layer resulting in solid/lipophilic core (78, 79). They can assume micelle-like structures to encapsulate drugs in their non-aqueous core, have high delivery rates and better endosomal escape and show low lipid accumulation in target organs. Lipid nanoparticles are composed of phospholipids, sterols and polyethylene glycol (80)-conjugated lipids, which protect the mRNA from nuclease degradation and immune responses and help in their cellular uptake (81). mRNAs encapsulated in LNPs have been trialled in animal models of methylmalonic acidaemia (82), acute intermittent porphyria (83), ornithine transcarbamylase (84) deficiency (85), arginase deficiency (86) and galactosaemia (87). Phase I/II clinical trials have been initiated in OTC patients to test the safety and efficacy of OTC mRNA formulated in lipid nanoparticles (76). These advancements in liposome-based cargo carriers will undoubtedly provide a platform in further development of gene therapy non-viral vector carriers.

### Preclinical development

Proof of concept of gene therapy has been achieved in many animal models recapitulating the human phenotype of IMDs: urea cycle defects, organic acidurias, maple syrup urine disease, phenylketonuria, tyrosinaemia type 1, glycogen storage disease type Ia, long-chain fatty acid oxidation disorders, homozygous familial hypercholesterolaemia, lipoprotein lipase deficiency, primary hyperoxaluria type I, progressive familial intrahepatic cholestasis, Wilson disease (4), Pompe disease (88), Gaucher disease (89), mucopolysaccharidosis (90, 91) and mitochondrial diseases such as mitochondrial neurogastrointestinal encephalomyopathy (92). For example, AAV vectors have successfully targeted the four most common urea cycle defects: OTC deficiency (93), citrullinaemia 1 (94), argininosuccinic aciduria (95) and arginase deficiency (96). It is noteworthy that the extrapolation of data for dose finding is often reliable from animal studies to clinical trials (28).

While most of these studies are using *in vivo* gene addition with non-integrating AAV vectors or *ex vivo* gene addition with integrative lentiviral vectors, some publications have shown proof of concept of *in vivo* integrative gene addition using a double AAV vector systems combining both a locus-specific nuclease-based integration system and the transgene to insert into the host genome, e.g. ZFNs in lysosomal storage diseases such as mucopolysaccharidosis types 1 (97) and 2 (98) or Piggybac transposase (99) in neonatal mouse model of OTC deficiency. Similarly, an innovative and successful strategy has been tested using CrispR/Cas9-mediated gene editing in the OTC-deficient mouse but highlighting the risk of off-target genome modifications (21).

### Ongoing clinical trials for IMD

Various clinical trials for IMD are currently planned, ongoing or completed, targeting mainly the brain or the liver. A non-exclusive list is provided in Table 2. Most trials are using either *ex vivo* gamma-retroviral or lentiviral vectors or *in vivo* AAV vectors. Examples illustrating these two main approaches are presented as follows.

**Table 2:** Indicative list of gene therapy clinical trials for inherited metabolic diseases in 2020.

Inherited metabolic diseases subgroup	Disease	Sponsor	Phase	Status	Vector	NCT number (*Clinicaltrials.gov*)
	Glycogen storage disease 1A	Ultragenyx	I/II	R	AAV8	NCT035117085
	Crigler-Najjar	Genethon-Selecta Bio	I/II	R	AAV	NCT03466463
	Ornithine transcarbamylase deficiency	University of Pennsylvania	I	T	Adeno- viral	NCT00004498
		Ultragenyx	I/II	R	AAV8	NCT02991144
Intermediary metabolism	Methylmalonic acidaemia	Moderna Therapeutics	I/II	R	Non viral	NCT03810690
	Propionic acidaemia	Moderna Therapeutics	I/II	A-NR	Non viral	NCT04159103
	Phenylketonuria	Homology Medicines	I/II	R	AAVH- SC15	NCT03952156
		National Taiwan University Hospital	I/II	T	AAV2	NCT01395641
	Aromatic L-amino acid decarboxylase deficiency	National Taiwan University Hospital	II	R	AAV2	NCT02926066
		National Institute of Health	I	R	AAV2	NCT02852213
Lipid metabolism	Homozygous Familial Hypercholesterolaemia	RegenX Bio	I/II	R	AAV	NCT02651675
		Sangamo Therapeutics	I/II	H	AAV6	NCT02702115
	Mucopolysaccharidosis 1	RegenX Bio	I	R	AAV9	NCT03580083
		Orchard Therapeutics/San Raffaele-Telethon Institute for Gene Therapy	I/II	R	LV	NCT03488394
	Mucopolysaccharidosis 2	Sangamo Therapeutics	I/II	H	AAV6	NCT03041324
		RegenX Bio	I/II	R	AAV9	NCT03566043
		Manchester University	I/II	R	LV	NCT04201405
		Lysogene	I/II	T	AAVrh10	NCT01474343
	Mucopolysaccharidosis 3A	Lysogene	I/II	R	AAVrh10	NCT03612869
		Abeona Therapeutics	I/II	R	AAV9	NCT02716246; NCT04088734
	Mucopolysaccharidosis 3B	Abeona Therapeutics	I/II	R	AAV9	NTC03315182
		Uniqure	I/II	T	AAV5	NCT03300453
	Mucopolysaccharidosis 6	Fondazione Telethon	I/II	R	AAV8	NCT03173521
		Audentes Therapeutics	I/II	A-NR	AAV8	NCT04174105
		Spark Therapeutics	I/II	A-NR	AAV	NCT04093349
Lysosomal storage diseases	Pompe disease	Florida University	I/II	T	AAV1	NCT00976352
		Florida University	I	R	AAV9	NCT02240407
	Danon disease	Rocket Pharmaceuticals	I	R	AAV9	NCT03882437
		Sangamo Therapeutics	I/II	R	AAV6	NCT04046224
	Fabry disease	Freeline Therapeutics	I/II	R	AAV	NCT04040049
		AvroBio	I/II	R	LV	NCT03454893
	Ceroide lipofuscinosis 6	Amicus Therapeutics	I/II	A-NR	AAV9	NCT02725580
	Ceroide lipofuscinosis 3	Amicus Therapeutics	I/II	R	AAV9	NCT03770572
		Cornell University	I	A-NR	AAV.rh10	NCT01161576
	Ceroide lipofuscinosis 2	Cornell University	I	A-NR	AAV2	NCT00151216
		Cornell University	I/II	A-NR	AAV.rh10	NCT01414985
	GM1 Gangliosidosis	National Human Genome Research Institute	I/II	R	AAV9	NCT03952637
	Metachromatic leukodystrophy	Orchard Therapeutics/San Raffaele-Telethon Institute for Gene Therapy	I/II	R	LV	NTC03392987
		Shenzhen University	I/II	R	LV	NCT02559830
	Gaucher type 1	AvroBio	I/II	R	LV	NCT04145037
		Bluebird Bio	II/III	A-NR	LV	NCT01896102
Peroxisomal disorders	X-linked childhood cerebral adrenoleukodystrophy	Bluebird Bio	III	A-NR	LV	NCT03852498
		Shenzhen Second People's Hospital	I/II	R	LV	NCT0372755

Information gathered from *clinicaltrials.gov* accessed on 22/01/2020. A-NR : active-not recruiting; H : on hold ; NR : not recruiting ; R : recruiting ; T : terminated

Information gathered from clinicaltrials.gov accessed on 22 January 2020.

A-NR, active-not recruiting; H, on hold; NR, not recruiting; R, recruiting; T, terminated.

### *Ex vivo* lentiviral gene therapy for cerebral inherited disease: example of X-linked childhood cerebral adrenoleukodystrophy

X-linked adrenoleukodystrophy is the most common peroxisomal disorder caused by mutations in the *ABCD1* (ATP-binding cassette subfamily D member 1 protein) gene. This protein enables the uptake of very long-chain fatty acids in the peroxisome to undergo degradation. In the most severe form with childhood-onset cerebral X-linked adrenoleukodystrophy, patients will develop normally until the age of 5–7 years when they will present a rapidly progressing neuroregression with loss of cognitive, motor, visual and auditive skills leading to vegetative state and death in less than 5 years. The standard of care is currently allogeneic HSC at early stage of the disease, enabling an arrest of the cerebral demyelination and disease progression. An *ex vivo* lentiviral vector encoding the *ABCD1* gene transfecting autologous human CD34+ cells was initially developed by Cartier et al. and showed stabilisation of the disease in two boys with no HLA-matched donors with an efficiency similar to HSC (10). This was followed by a multicentre, phase II/III clinical trial conducted by BlueBird Bio (NCT01896102) with a vector Lenti-D similar to the one used in treating the first two patients. Preliminary results showed that 88% of the 17 patients enrolled had only a minimal residual disease at a median follow-up of »30 months. The gene therapy showed measurable ALD protein in all patients, a polyclonal haematopoietic reconstitution with no evidence of preferential integration and clonal outgrowth and no graft-versus-host disease (100). This programme has been granted breakthrough therapy designation by the Food and Drug Administration (FDA) and a phase III trial (NCT03852498) has started in 2019 (101). This programme illustrates the major clinical benefits of this gene therapy approach for neurodegenerative diseases.

### *In vivo* AAV gene therapy for inherited liver disease: example of haemophilia B

Haemophilia B is a X-linked inherited severe bleeding disorder caused by factor IX (FIX) deficiency with a frequency of 1 in 30,000 male live births. The measurable plasma levels of FIX stratify the clinical severity: severe (<1%), moderate (1–5%) and mild (5–40%). The standard of care relies on injections of recombinant FIX, an expensive therapy for public healthcare systems with an annual cost of US$300,000 (102). As a minimal increase of plasma FIX can dramatically improve the clinical phenotype, various programmes have targeted haemophilia B. A phase I/II trial sponsored by Sparks Therapeutics and Pfizer (NCT02484092) has used an AAV vector expressing an improved variant of FIX cDNA known as Padua variant with a gain-of-function activity enabling a 5–10 times increase of FIX activity levels under the transcriptional expression of the liver-specific hAAT promoter. This vector was administered by a single peripheral intravenous injection at a dose of 5 ´ 10^11^ vector genomes per kilogram body weight. Preliminary results showed an increase in FIX from <1% at baseline at 1 week after infusion, reaching a plateau at 3 months post-injection and remaining stable over 1 year with a mean sustained FIX activity at 34 ± 18.5%. The annual bleeding rate was significantly reduced (from 11.1 to 0.4 events per year; *p* = 0.02) and 8 out of 10 patients managed to stop infusion of recombinant FIX. No patient experienced any severe adverse event. Only two patients presented asymptomatic increase of transaminases, well controlled by an oral course of corticosteroids (103). Increase of transaminases has been observed in the weeks following liver-targeted AAV gene therapy and recognised as a T-cell-mediated immune response against the capsid causing apoptosis of transduced hepatocytes. This adverse event, which usually happens in 3 months following the AAV injection, is often well controlled by oral corticosteroids but needs to be carefully monitored to prevent a loss of the benefit of the gene therapy (4). This haemophilia B programme has received breakthrough therapy designation by the FDA. A phase III trial is currently recruiting led by Pfizer (NCT03861273).

### Approved gene therapy products for IMD

Glybera is an AAV1-based vector carrying the lipoprotein lipase transgene, which has shown a reduction in acute pancreatitis episodes in patients with lipoprotein lipase deficiency. This product commercialised by Uniqure was the first gene therapy product approved by the European Medicine Agency (EMA) in 2012. Priced at US$1.1 million per patient, this treatment was administered only to one patient and Uniqure decided to withdraw the product from the market in 2017 due to economic considerations (38). Strimvelis is an *ex vivo* gene therapy approach using a gamma-retroviral vector containing the human adenosine deaminase (*ADA*) gene developed by GlaxoSmithKline to target ADA-SCID and priced US$665,000 (4). Luxturna is an AAV-based vector carrying the *RPE65* gene to treat Leber congenital amaurosis. Spark Therapeutics received market authorisation from the FDA and the EMA in 2017 and 2018, respectively, becoming the first FDA-approved gene therapy product and priced at US$425,000 per eye (38).

## Conclusion

Gene therapies have revolutionised the last half century from science fiction to the first commercialised products. New delivery strategies are constantly in development or refinement to achieve the most efficient and safest approach enabling with a single injection of gene therapy vector, a lifelong cure for severe IMDs. This novel therapy option will find its place among other available therapies, alone or in combination if necessary. Many open questions remain, especially about building an adequate economic model to enable affordable gene therapy products for healthcare systems and the long-term efficacy and safety of these novel therapies. Currently, research is designing novel genotype-specific molecular therapies that aim to become patient-specific and personalised gene therapy approaches to correct the phenotype of genetic diseases more efficiently.
